# The Association Between Cardiovascular Autonomic Function and Changes in Kidney and Myocardial Function in Type 2 Diabetes and Healthy Controls

**DOI:** 10.3389/fendo.2021.780679

**Published:** 2021-12-13

**Authors:** Jens Christian Laursen, Ida Kirstine B. Rasmussen, Emilie H. Zobel, Philip Hasbak, Bernt Johan von Scholten, Lene Holmvang, Rasmus S. Ripa, Christian S. Hansen, Marie Frimodt-Moeller, Andreas Kjaer, Peter Rossing, Tine W. Hansen

**Affiliations:** ^1^ Complications Research, Steno Diabetes Center Copenhagen, Herlev, Denmark; ^2^ Department of Clinical Medicine, Faculty of Health and Medical Sciences, The University of Copenhagen, Copenhagen, Denmark; ^3^ Novo Nordisk, Bagsvaerd, Denmark; ^4^ Department of Clinical Physiology, Nuclear Medicine & PET and Cluster for Molecular Imaging, Rigshospitalet, Copenhagen, Denmark; ^5^ Department of Cardiology, Rigshospitalet, Copenhagen, Denmark

**Keywords:** type 2 diabetes, kidney function, myocardial function, atheroclerosis, PET-CT, cardiovascular autonomic diabetic neuropathy (CADN)

## Abstract

**Background:**

The mechanisms linking cardiovascular autonomic neuropathy, diabetic kidney disease and cardiovascular mortality in type 2 diabetes are widely unknown. We investigated the relationship between baseline cardiovascular autonomic function and changes in kidney and myocardial function over six years in patients with type 2 diabetes and healthy controls.

**Methods:**

*Post-hoc* analysis of a cohort study in 24 patients with type 2 diabetes and 18 healthy controls. Baseline determinants were cardiovascular autonomic reflex tests (heart rate response to: standing (30:15); deep breathing (E:I); and the Valsalva test) and time- and frequency-domain heart rate variability indices. Outcomes were changes in estimated glomerular filtration rate (eGFR), albuminuria, myocardial flow reserve (MFR) measured by cardiac ^82^Rb Positron emission tomography computed tomography (PET/CT), and coronary artery calcium score (CACS).

**Results:**

Mean age at inclusion was 61 ± 10 years and 36% were female. Mean follow up time was 6 ± 0 years. A lower response in heart rate to the Valsalva test (corresponding to weaker autonomic function) was associated with a larger decline in eGFR (p=0.04), but not significantly after adjustment for sex, baseline age, smoking status, systolic blood pressure, heart rate, HbA_1c_, body mass index and baseline eGFR (p=0.12). A higher baseline response in heart rate to standing (30:15) was associated with a larger decline in myocardial flow reserve in the unadjusted analysis (p=0.02) and after adjustment (p=0.02). A higher response in heart rate to the Valsalva maneuver was associated with a larger increase in CACS (p = 0.02), but the association became insignificant after adjustment (p = 0.16).

**Conclusion:**

A lower response in heart rate to the Valsalva test was associated with a larger decline in kidney function, indicating that autonomic dysfunction may predict future loss of kidney function. However, we did not find any association between lower values in cardiovascular autonomic function at baseline and a worsening in albuminuria, myocardial function, or atherosclerotic burden.

## Introduction

Cardiovascular autonomic neuropathy (CAN) is an overlooked and frequent diabetic complication seen in up to 65% of persons with type 2 diabetes (1). CAN has been suggested to promote the progression of diabetic kidney disease and is an independent risk factor for cardiovascular mortality (1–3). The mechanisms linking CAN, diabetic kidney disease and cardiovascular mortality are widely unknown.

The role of CAN in the progression of kidney and coronary disease in diabetes have been investigated in various studies. An association between presence of CAN and faster progression in diabetic kidney disease has been demonstrated both in type 1 diabetes (3) and in type 2 diabetes (4, 5). A reduced myocardial flow reserve is a risk factor for cardiac death, non-fatal myocardial infarction (6) and independently associated with presence of CAN in type 2 diabetes (7). However, we could not find studies investigating the association between cardiovascular autonomic function and longitudinal changes in myocardial flow reserve in type 2 diabetes. Another cardiovascular measure associated with CAN in diabetes is the coronary artery calcium score, an established marker of coronary atherosclerosis (8–11). Only one study was found investigating the effect of CAN on changes in the coronary artery calcium score in type 1 diabetes, which could not demonstrate an association after two years of follow up (12). We found no studies investigating the association between CAN and changes in kidney and myocardial function concurrently.

We recently demonstrated that the myocardial flow reserve was lower, and the coronary artery calcium score was higher in type 2 diabetes as compared with healthy controls (13). In this *post hoc* analysis in the same population, we investigated the association between cardiovascular autonomic function at baseline and changes over six years in outcomes reflecting kidney function, myocardial function and atherosclerotic burden. We hypothesized that lower measures of cardiovascular autonomic function at baseline would be associated with a larger decline in kidney function and myocardial flow reserve and a larger increase in coronary artery calcium score.

## Methods

### Design

*Post hoc* analysis of a cohort study in type 2 diabetes and healthy controls studying temporal changes in myocardial function in patients with type 2 diabetes and non-diabetic controls (13). The study was performed in compliance with the Declaration of Helsinki. All participants gave informed written consent, and the study protocol was approved by a regional research ethics committee (H-19024534).

### Study Population

**Figure 1:**
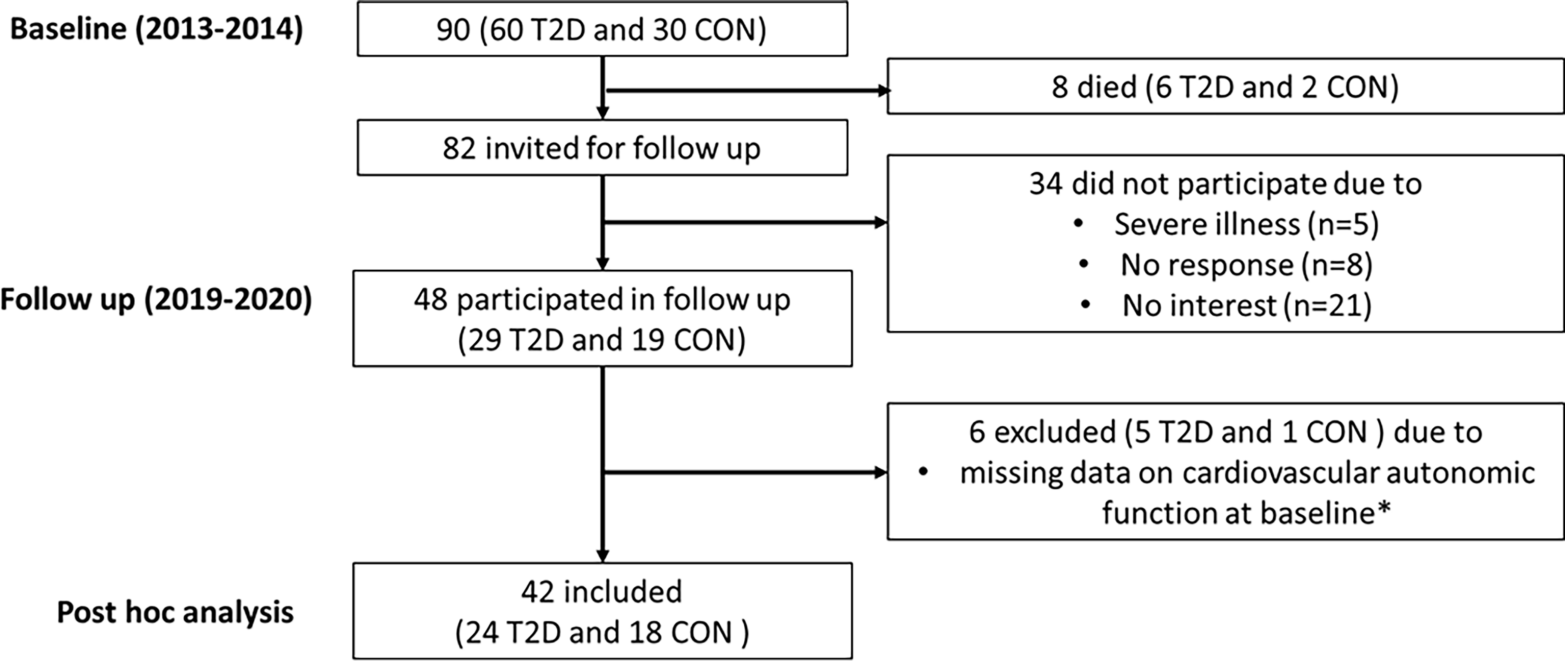
Flowchart for participants. T2D, Type 2 diabetes; CON, Healthy control. *Due to lack of compliance or malfunction of the equipment at the time of measurement.

In 2013-2014, 60 persons with type 2 diabetes and 30 age- and sex-matched healthy controls were recruited at Steno Diabetes Center Copenhagen (7). In the type 2 diabetes group, 30 had normoalbuminuria (<30mg/24h) and 30 had albuminuria (≥30mg/24h). Participants were between 35 and 80 years of age and free of overt cardiovascular disease. In 2019-2020, all living participants (n=82) were invited for a follow up visit. A total of 48 participants from the original cohort participated. Reasons for not participating were severe illness (n=5), no response (n=8) and not interested (n=21). Cardiovascular autonomic function data was missing for six participants (5 with type 2 diabetes and 1 control) due to lack of compliance or malfunction of the equipment at the time of measurement. Thus, the present *post hoc* analysis includes 42 participants (24 with type 2 diabetes and 18 controls) who had a complete dataset on cardiovascular autonomic function at baseline and on the study outcomes at baseline and follow up (**Figure 1**).

### Baseline Measurements

Laboratory variables included HbA_1c_, lipid profile and plasma creatinine measured by standard methods. Upper arm blood pressure was measured in sitting position on the right arm after 10-minutes rest with a validated blood pressure monitor and the mean of three consecutive measurements was calculated.

### Explanatory Variables at the Baseline Visit

Participants were advised to abstain from hard physical activity 24 hours before the examination. All tests of cardiovascular autonomic function were performed between 8:00 A.M. and 2:00 P.M. in a quiet examination room. Three standard cardiovascular autonomic reflex tests (CARTs) and 5-min resting heart rate recordings for calculating time- and frequency-domain heart rate variability indices were obtained once (3, 14). Parameters of cardiovascular autonomic function were obtained using the Vagus Device (Medicus Engineering, Aarhus, Denmark) (15). The CARTs included the heart rate response to: 1) standing (30:15); 2) deep breathing (E:I); and 3) the Valsalva test. The 30:15 ratio, E:I ratio and Valsalva tests are measures mainly of parasympathetic function (1). The CARTs were evaluated using age-related reference intervals (16). CAN was defined using the American Diabetes Association criteria and participants with two or three pathological CARTs were classified as having CAN (17). From the 5-min resting heart rate recordings, time-domain heart rate variability indices were derived: the standard deviation of normal-to-normal (SDNN) intervals and the root mean square of successive differences (RMSSD). Frequency-domain heart rate variability indices were calculated using fast Fourier transformation and included low frequency (LF) power (0.04–0.15 Hz), high frequency (HF) power (0.15–0.4 Hz), and the total (Total) power (0.4 Hz). The ratio of LF-to-HF (LF/HF Ratio) was also calculated. Where RMSSD and HF power reflects parasympathetic activity, the SDNN, LF power and Total power are measures of combined sympathetic and parasympathetic activity.

### Outcome Variables at the Baseline and Follow Up Visits

The estimated glomerular filtration rate (eGFR) was calculated by the CKD-EPI equation (18). The urine albumin excretion was measured at the baseline visit in two 24 hours urine collections and estimated as the urine albumin excretion rate and measured at the follow up visit in three consecutive morning urine samples and estimated as the urine albumin creatinine ratio. Cardiac ^82^Rb Positron emission tomography computed tomography (PET/CT) imaging provides a quantitative measurement of the myocardial perfusion (ml/min/g of tissue) during stress and rest. This enables calculation of the myocardial flow reserve (19). The cardiac ^82^Rb PET/CT scans were performed using a hybrid PET/CT scanner in 3D mode (Siemens Biograph mCT 128, Siemens, Munich, Germany) after administration of 1,100 MBq ^82^Rb (CardioGen-82, Bracco Diagnostics, Monroe Township, NJ, USA). The scans were performed at rest and at stress (adenosine infusion to induce maximum myocardial hyperemia). The scans were performed with similar equipment, protocol and analyzed using identical software (syngo.*via* VB20A) by the same single observer at the baseline and follow up visits. The coronary artery calcium score was calculated by the method described by Agatston et al. as the sum of coronary artery calcium content in the three main coronary arteries (20) using semi-automated commercially available software (Syngovia 4.0).

### Statistical Analysis

Clinical characteristics of the participants are presented as n (%), mean ± standard deviation (SD) or if skewed distributed, as medians with interquartile range [quartile 1 to quartile 3]. The variables with skewed distributions were log transformed (natural logarithm) before analyses and normal distribution was obtained. A value of 1 was added to the coronary artery calcium score before log transformation, since the skewed distribution included values of zero. We applied the Chi-squared test or Fisher’s exact test for categorical data and unpaired Student’s t-tests for continuous variables to analyze differences between the participants with type 2 diabetes and the healthy controls. Cross-sectional associations in the total population were assessed in the baseline study as already publiched (7). We repeated these analyses in the present sub-population, using unadjusted linear regression models to evaluate the association between baseline cardiovascular autonomic function parameters and baseline levels of outcomes. Next, to control for possible confounders, we adjusted the significant associations for sex, baseline age, smoking, systolic blood pressure, heart rate, HbA_1c_ and body mass index. Change from baseline to follow up was analyzed using paired Student’s t-tests. Changes in the outcomes were expressed per year as the change from the baseline to the follow up visit, divided by the individual follow up time in years. Unadjusted and adjusted linear regression models were applied to assess associations between the baseline cardiovascular autonomic function parameters (30:15, E:I, Valsalva, log (SDNN), log (RMSSD), log (LF), log (HF), log (Total) and log (LF/HF Ratio)) and yearly changes in the outcomes. Adjusment included sex, baseline age, smoking, systolic blood pressure, heart rate, HbA_1c_, body mass index and the baseline value of the given outcome. All participants were pooled in the linear regression analyses and standardized regression coefficients are reported. We used SAS Enterprise Guide, version 7.15 for analyses. Two-sided p values < 0.05 were considered statistically significant. Due to the exploratory nature of this *post hoc* analysis, we did not perform a sample size calculation for this study.

## Results

### Baseline Characteristics

Age and sex distributions were equal in the 24 persons with type 2 diabetes and the 18 healthy controls. The persons with type 2 diabetes had a higher body mass index and heart rate and lower LDL and total cholesterol compared with controls. The participants with type 2 diabetes had a median [Q1 to Q3] diabetes duration of 11 [5 to 19] years, mean HbA_1c_ of 53 ± 10 mmol/mol, eGFR of 79 ± 22 ml min^−1^ 1.73 m^−2^ and 11 (46%) had albuminuria (**Table 1**).

**Table 1:** Baseline characteristics.

	T2D	Control	*p*
Numbers of participants	24	18	
Age (years)	62 ± 10	59 ± 9	0.34
Women	7 (29%)	8 (44%)	0.31
Non-smokers	20 (83%)	15 (83%)	1
Diabetes duration (years)	11 [5 to 19]	–	–
Albuminuria	11 (46%)	0 (0%)	–
Body mass index (kg/m^2^)	31.3 ± 5.0	24.2 ± 3.4	<0.001
Heart rate (beats minute ^-1^)	68 ± 9	59 ± 8	0.003
Systolic blood pressure (mmHg)	136 ± 15	124 ± 13	0.02
Diastolic blood pressure (mmHg)	79 ± 8	74 ± 8	0.09
HbA_1c_ (%)	7.0 ± 0.9	5.4 ± 0.2	–
HbA_1c_ (mmol/mol)	53 ± 10	36 ± 2	–
LDL cholesterol (mmol/L)	2.2 ± 0.8	3.1 ± 0.7	<0.001
Antihypertensive treatment	23 (96%)	0 (0%)	–
**Cardiovascular autonomic function**			
Cardiovascular Autonomic Neuropathy*	1 (4%)	0 (0%)	–
30:15 (Lying to standing ratio)	1.2 ± 0.1	1.3 ± 0.2	0.05
E:I (Expiration to inspiration ratio)	1.1 ± 0.1	1.2 ± 0.1	0.02
Valsalva (Before to after Valsalva ratio)	1.5 ± 0.2	1.7 ± 0.4	0.05
SDNN (ms)	23 [20 to 28]	41 [32 to 54]	<0.001
RMSSD (ms)	14 [10 to 19]	29 [24 to 44]	0.001
LF (ms^2^)	46 [24 to 67]	207 [123 to 331]	<0.001
HF (ms^2^)	22 [13 to 41]	93 [62 to 180]	0.001
Total (ms^2^)	179 [126 to 238]	729 [301 to 165]	<0.001
LF/HF ratio	1.4 [0.7 to 3.7]	1.9 [1.2 to 4.2]	0.34

Data are n (%), mean ± SD, or median [Q1 to Q3]. P-values for differences between participants with type 2 diabetes (T2D) and controls were calculated using the Chi-squared test or Fisher’s exact test for categorical data and Student’s t-tests for quantitative data. Albuminuria was defined as a urine albumin excretion rate > 30 mg hours^-1^. 30:15; E:I and Valsalva are heart rate variability response to cardiovascular reflex tests; 30:15, lying to standing; E:I, expiration to inspiration; Valsalva, The Valsalva maneuver; SDNN, standard deviation of the normal-normal interval; RMSSD, root mean square of the successive differences; LF, low frequency power; HF, high frequency power; Total, total power. *Presence of CAN was defined as two or three pathological cardiovascular autonomic reflex tests.

### Cardiovascular Autonomic Function at Baseline

All measures of cardiovascular autonomic function at baseline, except the 30:15, the Valsalva and the LF/HF ratio, were lower in the participants with type 2 diabetes compared with the healthy controls. Only one participant (with type 2 diabetes) had CAN (**Table 1**). Baseline associations between cardiovascular autonomic function and measures of heart and kidney function are shown in **Table S1**. A higher (more beneficial) 30:15 ratio was associated with a higher eGFR. Higher values of all measures of cardiovascular autonomic function, except the E:I ratio, RMSSD, high frequency power and the LF/HF ratio, were associated with lower urine albumin excretion rate. Higher values of all measures of cardiovascular autonomic function, except the Valsalva ratio, High Frequency power and LF/HF ratio, correlated with higher baseline myocardial flow reserve. Finally, higher values of all cardiovascular autonomic function parameters, except the LF/HF ratio, were associated with lower baseline coronary artery calcium score. None of the associations at baseline remained significant after confounder adjustment (**Table S1**).

### Longitudinal Changes in Outcomes

Baseline and follow-up values of outcomes are shown in **Table 2**. The mean ± SD follow up time was 6.2 ± 0.2 years for participants with type 2 diabetes and 6.3 ± 0.2 years for the healthy controls. The mean change per year in eGFR was -1.2 ± 1.3 ml min^−1^ 1.73 m^−2^ for participants with type 2 diabetes and -0.7 ± 0.8 ml min^−1^ 1.73 m^−2^ for healthy controls, with no significant difference between groups (p=0.16). The median change per year in urine albumin was -0.9 [-7.3 to 0.3] mg g^-1^ for the type 2 diabetes group and -0.2 [-0.5 to 0.1] mg g^-1^ for the healthy controls, with no significant difference between groups (p=0.34). There was no difference between groups in the yearly change in myocardial flow reserve. The median change per year in coronary artery calcium score was significantly larger in the type 2 diabetes group (p=0.003) (**Table 2**).

**Table 2:** Baseline and follow up values of outcomes.

	T2D	Control	*p*
Number of participants	24	18	
**Baseline values**			
Estimated Glomerular Filtration Rate (ml min^−1^ 1.73 m^−2^)	79 ± 22	88 ± 11	0.12
Urine albumin excretion rate (mg 24 hours^-1^)	27 [7 to 146]	6 [5 to 6]	0.04
Myocardial Flow Reserve	2.7 ± 0.7	3.4 ± 0.7	0.01
Coronary Artery Calcium Score	205 [25 to 470]	0 [0 to 54]	0.01
**Follow up values**			
Estimated Glomerular Filtration Rate (ml min^−1^ 1.73 m^−2^)	71 ± 24	83 ± 10	0.047
* p vs. baseline*	<0.001	0.002	
Urine albumin creatinine ratio (mg g^-1^ creatinine)	9 [5 to 157]	4 [3 to 7]	0.03
* p vs. baseline*	0.04	0.04	
Myocardial Flow Reserve	2.5 ± 0.6	3.2 ± 0.9	0.003
* p vs. baseline*	0.10	0.52	
Coronary Artery Calcium Score	700 [162 to 1025]	0 [0 to 82]	0.003
* p vs. baseline*	<0.0001	0.03	
**Change per year**			
Estimated Glomerular Filtration Rate (ml min^−1^ 1.73 m^−2^)	-1.2 ± 1.3	-0.7 ± 0.8	0.16
Urine albumin (mg g^-1^ creatinine)	-0.9 [-7.3 to 0.3]	-0.2 [-0.5 to 0.1]	0.34
Myocardial Flow Reserve	-0.04 ± 0.11	-0.02 ± 0.13	0.62
Coronary Artery Calcium Score	55 [10 to 141]	0 [0 to 8]	0.003

Data are mean ± SD and median [Q1 to Q3]. P-values for differences between participants with type 2 diabetes (T2D) and controls were calculated using unpaired Student’s t-tests.P-values for difference from baseline to follow up was calculated using paired Student’s t-tests.

### Associations Between Cardiovascular Autonomic Function at Baseline and Changes in Outcomes

Associations between cardiovascular autonomic function at baseline and changes in heart and kidney function are shown in **Table 3**. A lower response in heart rate to the Valsalva test, reflecting impaired cardiovascular autonomic function, was associated with a larger decline in eGFR in the unadjusted model (β per 1 SD = 0.37 ml min^−1^ 1.73 m^−2^ increase per year; p=0.04), but not after adjustment (p=0.12). No other associations between cardiovascular autonomic function at baseline and changes in eGFR were found. No associations with changes in urine albumin were found. A higher response in heart rate to standing (30:15), reflecting better cardiovascular autonomic function, was associated with a larger decline in myocardial flow reserve in the unadjusted model (β per 1 SD = 0.04 decrease per year; p=0.02) and after adjustment (p=0.02). No other associations between cardiovascular autonomic function at baseline and changes in myocardial flow reserve were demonstrated. A higher response in heart rate to the Valsalva maneuver was associated with a larger increase in coronary artery calcium score (β per 1 SD = 0.34 increase per year; p = 0.02), but the association became insignificant after adjustment (p = 0.16). There were no other associations between cardiovascular autonomic function at baseline and changes in coronary artery calcium score (**Table 3**).

**Table 3:** Associations between cardiovascular autonomic function at baseline and changes in heart and kidney function.

Variable	eGFR		Urine Albumin		Myocardial Flow Reserve		Coronary Artery Calcium Score	
	β	*p*	β	*p*	β	*p*	β	*p*
30:15	-0.06	0.75	0.01	0.84	**-0.04**	**0.02**	-0.01	0.92
E:I	0.25	0.18	0.02	0.57	-0.01	0.44	0.19	0.19
Valsalva	**0.37**	**0.04**	0.02	0.54	0.00	0.94	**0.34**	**0.02**
SDNN	0.29	0.10	0.02	0.40	0.01	0.69	0.23	0.08
RMSSD	0.12	0.51	0.02	0.51	0.02	0.36	0.16	0.25
LF	0.19	0.29	0.04	0.13	-0.01	0.50	0.19	0.16
HF	0.14	0.45	0.00	0.99	0.01	0.44	0.15	0.30
Total	0.19	0.30	0.02	0.58	0.00	0.91	0.24	0.08
LF/HF Ratio	0.06	0.74	0.06	0.05	-0.03	0.07	0.08	0.59

Data are standardized β-estimates and p-values from unadjusted linear regression models with the yearly change in the outcome (myocardial flow reserve, log (coronary artery calcium score), eGFR and log (urine albumin) as outcome and the cardiovascular autonomic function parameter at baseline [30:15; E:I; Valsalva; Log (SDNN); Log (RMSSD); Log (LF); Log (HF); Log (Total); Log (LF/HF ratio)] as the exposure. The natural logarithm was applied for the log transformation. eGFR, estimated glomerular filtration rate; 30:15; E:I and Valsalva are heart rate variability response to cardiovascular reflex tests; 30:15, lying to standing; E:I, expiration to inspiration; Valsalva, The Valsalva maneuver; SDNN, standard deviation of the normal-normal interval; RMSSD, root mean square of the successive differences; LF, low frequency power; HF, high frequency power; Total, total power; Significant associations are marked with bold.

### Sensitivity Analyses

Changes in medical therapy from baseline to follow up are shown in **Table S2**. In the type 2 diabetes group, a total of 21 (88%) had changes in their diabetes therapy from baseline to follow up (insulin; sodium-glucose cotransporter 2 inhibitor (SGLT2i); glucagon-like peptide-1 receptor agonist (GLP1-RA); metformin) which might have influenced our results. To take this into account, we adjusted our analyses further for change in diabetes therapy from baseline to follow up (yes vs. no). Results were confirmatory. To account for the influence on beta-blockers on the cardiovascular autonomic function tests, we performed a sensitivity analysis, excluding the two participants treated with a beta-blocker at baseline. Results were confirmatory.

## Discussion

We observed that a lower response in heart rate to the Valsalva test was associated with a larger decline in kidney function over six years. We could not demonstrate any associations between lower cardiovascular autonomic function and a larger decline over six years in myocardial flow reserve as hypothesized, but we did observe an association in the opposite direction with a higher response in heart rate to standing (30:15). Finally, there was no association between cardiovascular autonomic function at baseline and changes in coronary artery calcium score.

The presence of cardiovascular autonomic dysfunction has been associated with faster progression of kidney disease in type 2 diabetes. This was shown by Yun and colleagues in a longitudinal study including 755 persons with type 2 diabetes and 9.6 years of follow up, and by Tahrani et al. in a longitudinal study including 204 persons with type 2 diabetes followed for 2.5 years (4, 5). We observed an association between a lower response in heart rate to the Valsalva test and a larger decline in eGFR, which was in line with our hypothesis and with these studies. The mechanisms are largely unknown, but renal hemodynamic changes associated with autonomic dysfunction (e.g. higher blood pressure during the night) could play a role (21). Another possibility is that parasympathetic dysfunction and a sympathetic overdrive promotes renal destruction, as already suggested in type 1 diabetes (22).

Measures of cardiovascular autonomic function were lower at baseline in the type 2 diabetes group than in the healthy controls, indicating some degree of autonomic dysfunction, but only one participant (4%) was diagnosed with CAN based on pathologic response in CARTs. We expected a prevalence in the range of 44% to 65% in a type 2 diabetes population with this mean age and diabetes duration (1, 3). This very low prevalence of cardiovascular autonomic dysfunction could have influenced our results. Lower values in the response in heart rate to standing (30:15), deep breathing (E:I) and the Valsalva test and lower values of the time- and frequency-domain heart rate variability indices (SDNN, RMSSD, LF power, HF power and Total power) were all associated with a lower myocardial flow reserve in the previously published cross-sectional baseline study (7) and similar associations were demonstrated at baseline in this subpopulation. Thus, we expected an association between lower cardiovascular autonomic function at baseline and a larger decline in myocardial flow reserve, but the only association we found was between a higher response in heart rate to standing (30:15) and a larger decline in myocardial flow reserve. The estimated decline in myocardial flow reserve of 0.04 per year per SD increase in the response in heart rate was however quite small compared to the mean difference in myocardial flow reserve of 0.7 (95%CI 0.2-1.1) between the groups at baseline (considered as a clinically relevant difference). Regression to the mean could in part explain this association. Finally, survival bias might be an important limitation to this study and might have influenced our results as discussed below. We are not aware of previous studies investigating associations between cardiovascular autonomic function and changes in myocardial flow reserve and further studies in larger populations are warranted.

The coronary artery calcium score increased during follow up in both groups and significantly more in the type 2 diabetes group. Lower autonomic cardiovascular function was associated with a higher atherosclerotic burden at baseline (7), but not with faster progression during six years of follow up. This is in line with a previous study in type 1 diabetes (12).

### Limitations

The investigated population was small, and this introduces a risk of false negative findings due to lack of power. Only 42 out of the original 90 persons participated in the follow up. The participants in the follow-up examination were younger, had a higher myocardial flow reserve, better kidney function and a lower coronary artery calcium score at the baseline examination compared with non-participants (13). The baseline characteristics of the full original cohort of 90 subjects and the follow up cohort of 42 subjects are presented in **Table S3**. This introduces a considerable risk of selection- and survival bias which weakens our chance to find associations. Thus, our results should be interpreted as exploratory and with this limitation in mind. When conducting a large number of tests as we have done there is a risk of type 1 errors. The myocardial flow reserve did not change in neither of the groups during follow up which makes it difficult to study associations between autonomic function and change in myocardial flow reserve. It is a limitation that participants were not abstinent from medication before measurements of cardiovascular autonomic function at baseline. Moreover, we did not evaluate the cardiovascular autonomic function at follow up with the Vagus device as in the baseline examination and are therefore not able to relate changes in cardiovascular autonomic function to the outcomes.

## Conclusion

With this *post hoc* analysis we are the first to investigate the longitudinal associations between cardiovascular autonomic function at baseline and changes in kidney function, myocardial function, and atherosclerotic burden over six years of follow up. A lower response in heart rate to the Valsalva test was associated with a larger decline in kidney function, indicating that autonomic dysfunction may be a risk factor for future loss of kidney function. We unexpectedly found no association between lower cardiovascular autonomic function at baseline and larger decline in the myocardial flow reserve. Furthermore, we found no association between baseline cardiovascular autonomic function measures and changes in the atherosclerotic burden. Further studies investigating the relation between cardiovascular autonomic function and changes in kidney and myocardial function in diabetes are needed.

## Data Availability Statement

Anonymized data can be obtained from the corresponding author upon reasonable request. Necessary data protection agency and ethical committee approvals must be provided in compliance with relevant legislation.

## Ethics Statement

The studies involving human participants were reviewed and approved by The Regional Research Ethics Committee (Central Region) (H-19024534). The patients/participants provided their written informed consent to participate in this study.

## Author Contributions

JL drafted the manuscript. JL, IR, EZ, PH, BS, LH, RR, CH, MF-M, AK, PR, and TH analyzed and interpreted the research. JL, IR, EZ, RR, AK, PR, and TH conceived and designed the research. JL and TH performed the statistical analysis. JL, IR, EZ, PH, BS, RR, and AK did measurements. TH supervised the study. EZ, RR, AK, PR, and TH obtained the funding. JL is the guarantor of this work and, as such, had full access to all the data in the study and takes responsibility for the integrity of the data and the accuracy of the data analysis. All authors contributed to the interpretation of the results, reviewed and edited the manuscript and approved the final version of the manuscript.

## Funding

The authors acknowledge the support from the Novo Nordisk Foundation (grant number NNFOC0013659): PROTON: Personalizing Treatment of Diabetic Nephropathy; The Novo Nordisk Foundation (grant number NNF19OC0054674) ‘Deep phenotyping of the heart with advanced imaging modalities in Type 2 diabetes implications for pathophysiology and prognosis (DIA-HEART study)’, Skibsreder Per Henriksen, R. og Hustrus Fond and internal funding from the Steno Diabetes Center Copenhagen, Denmark and Rigshospitalet, Copenhagen, Denmark.

## Conflict of Interest

PR reports having received research grants from Astra Zeneca and Novo Nordisk and given lectures for Astra Zeneca, Mundipharma and Boehringer Ingelheim and has served as a consultant for Astra Zeneca, Bayer, Eli Lilly, Boehringer Ingelheim, Astellas, Gilead, Sanofi Aventis Vifor, and Novo Nordisk, all fees given to Steno Diabetes Center.

The remaining authors declare that the research was conducted in the absence of any commercial or financial relationships that could be construed as a potential conflict of interest.

## Publisher’s Note

All claims expressed in this article are solely those of the authors and do not necessarily represent those of their affiliated organizations, or those of the publisher, the editors and the reviewers. Any product that may be evaluated in this article, or claim that may be made by its manufacturer, is not guaranteed or endorsed by the publisher.
