# Supplementary material for: The Association Between Cardiovascular Autonomic Function and Changes in Kidney and Myocardial Function in Type 2 Diabetes and Healthy Controls
Source: Front Endocrinol (Lausanne). 2021 Dec 13;12:780679. doi: 10.3389/fendo.2021.780679 (PMC8710600; doi:10.3389/fendo.2021.780679)
Supplement: Supplementary file 1 [file Table_1.docx]

**Table S1.**  Associations between cardiovascular autonomic function and heart and kidney function at baseline

|  | **eGFR** | | **Urine Albumin** | | **Myocardial Flow Reserve** | | **Coronary Artery Calcium Score** | |
| --- | --- | --- | --- | --- | --- | --- | --- | --- |
| Variable | β | *p* | β | *p* | β | *p* | β | *p* |
| 30:15 | **5.74** | **0.04** | **-0.85** | **0.02** | **0.25** | **0.02** | **-1.32** | **0.02** |
| E:I | 3.73 | 0.21 | -0.54 | 0.16 | **0.27** | **0.01** | **-2.34** | **<0.001** |
| Valsalva | 4.84 | 0.10 | **-0.82** | **0.01** | 0.21 | 0.06 | **-1.42** | **0.01** |
| SDNN | 5.23 | 0.06 | **-0.76** | **0.03** | **0.28** | **0.01** | **-2.10** | **<0.001** |
| RMSSD | 4.20 | 0.14 | -0.66 | 0.07 | **0.25** | **0.02** | **-1.76** | **0.001** |
| LF | 2.56 | 0.37 | **-0.75** | **0.04** | **0.35** | **0.001** | **-1.94** | **<0.001** |
| HF | 3.03 | 0.29 | -0.66 | 0.07 | 0.17 | 0.14 | **-1.84** | **<0.001** |
| Total | 3.31 | 0.25 | **-0.91** | **0.01** | **0.32** | **0.003** | **-2.07** | **<0.001** |
| LF/HF Ratio | 2.78 | 0.33 | -0.28 | 0.47 | 0.08 | 0.49 | -0.70 | 0.23 |

Data are standardized β-estimates and *p-*values from unadjusted linear regression models with baseline values in heart and kidney function (myocardial flow reserve, log (coronary artery calcium score), eGFR and log (urine albumin) as outcome and the cardiovascular autonomic function parameter at baseline (30:15; E:I; Valsalva; Log (SDNN); Log (RMSSD); Log (LF); Log (HF); Log (Total); Log (LF/HF ratio)) as the exposure. The natural logarithm was applied for the log transformation. eGFR = estimated glomerular filtration rate. 30:15, E:I and Valsalva are heart rate variability response to cardiovascular reflex tests; 30:15 = lying to standing; E:I = expiration to inspiration; Valsalva = The Valsalva maneuver; SDNN = standard deviation of the normal-normal interval; RMSSD = root mean square of the successive differences; LF = low frequency power; HF = high frequency power; Total = total power.
