# Supplementary material for: The Association Between Cardiovascular Autonomic Function and Changes in Kidney and Myocardial Function in Type 2 Diabetes and Healthy Controls
Source: Front Endocrinol (Lausanne). 2021 Dec 13;12:780679. doi: 10.3389/fendo.2021.780679 (PMC8710600; doi:10.3389/fendo.2021.780679)
Supplement: Supplementary file 2 [file Table_2.docx]

| **Table S2.** Medication at baseline and follow up divided in participants with type 2 diabetes and healthy controls | | | | | | |
| --- | --- | --- | --- | --- | --- | --- |
|  | Type 2 diabetes | |  | Healthy control | |  |
|  | Baseline | Follow Up | *p* | Baseline | Follow Up | *p* |
| Numbers of participants | 24 | 24 | - | 18 | 18 | - |
| Antihypertensive treatment | 23 (96%) | 21 (96% | 0.30 | 0 (0%) | 0 (0%) | - |
| RAAS inhibition treatment | 22 (92%) | 21 (88%) | 0.64 | 0 (0%) | 1 (6%) | - |
| Treatment with diuretics | 14 (58%) | 15 (63%) | 0.77 | 0 (0%) | 1 (6%) | - |
| Calcium antagonist treatment | 11 (46%) | 9 (38%) | 0.56 | 0 (0%) | 1 (6%) | - |
| Beta-blocker treatment | 2 (8%) | 1 (4%) | 0.55 | 0 (0%) | 1 (6%) | - |
| Insulin treatment | 13 (54%) | 16 (67%) | 0.38 | 0 (0%) | 0 (0%) | - |
| Metformin treatment | 24 (100%) | 16 (67%) | <0.01 | 0 (0%) | 0 (0%) | - |
| SGLT2 inhibitor treatment | 0 (0%) | 8 (33%) | - | 0 (0%) | 0 (0%) | - |
| GLP-1 receptor agonist treatment | 0 (0%) | 13 (54%) | - | 0 (0%) | 0 (0%) | - |
| Lipid-lowering treatment | 23 (96%) | 22 (92%) | 0.55 | 0 (0%) | 2 (11%) | - |

Data are n (%). *P*-values for differences between baseline and follow up were calculated using the Chi-squared test or Fisher’s exact test. RAAS = renin-angiotensin-aldosterone system, SGLT2 = sodium-glucose cotransporter 2, GLP-1: glucagon-like peptide-1
