# Supplementary material for: The Association Between Cardiovascular Autonomic Function and Changes in Kidney and Myocardial Function in Type 2 Diabetes and Healthy Controls
Source: Front Endocrinol (Lausanne). 2021 Dec 13;12:780679. doi: 10.3389/fendo.2021.780679 (PMC8710600; doi:10.3389/fendo.2021.780679)
Supplement: Supplementary file 3 [file Table_3.docx]

| **Table S3.** Baseline characteristics of full original cohort vs. follow up cohort divided in type 2 diabetes and healthy controls | | | | |
| --- | --- | --- | --- | --- |
|  | Type 2 diabetes | | Healthy controls | |
|  | Full group | Follow up group | Full group | Follow up group |
| Numbers of participants | 60 | 24 | 30 | 18 |
| Age (years) | 63±9 | 62±10 | 60±10 | 59±9 |
| Women | 20 (33%) | 7 (29%) | 12 (40%) | 8 (44%) |
| Non-smokers | 40 (67%) | 20 (83%) | 23 (77%) | 14 (78%) |
| Diabetes duration (years) | 12 [6 to 18] | 11 [5 to 19] | - | - |
| Albuminuria | 30 (50%) | 11 (46%) | 0 (0%) | 0 (0%) |
| Body mass index (kg/m^2^) | 31.6±4.5 | 31.3±5.0 | 24.8±3.4 | 24.2±3.4 |
| Heart rate (beats minute ^-1^) | 72±12 | 68±9 | 61±9 | 6±0 |
| Systolic blood pressure (mmHg) | 138±19 | 136±15 | 132±14 | 131±14 |
| Diastolic blood pressure (mmHg) | 83±12 | 79±8 | 82±8 | 80±7 |
| HbA_1c_ (%) | 7.2±1.0 | 7.0±0.9 | 5.4±0.2 | 5.5±0.2 |
| HbA_1c_ (mmol/mol) | 56±11 | 53±10 | 36±2 | 36±2 |
| LDL cholesterol (mmol/L) | 2.2±0.9 | 2.2±0.8 | 3.4±0.7 | 3.1±0.7 |
| **Cardiovascular autonomic function** |  |  |  |  |
| Cardiovascular Autonomic Neuropathy* | 7 (13%) | 1 (4%) | 0 (0%) | 0 (0%) |
| 30:15 (Lying to standing ratio) | 1.1±0.1 | 1.2±0.1 | 1.2±0.2 | 1.3±0.2 |
| E:I (Expiration to inspiration ratio) | 1.1±0.1 | 1.1±0.1 | 1.2±0.2 | 1.2±0.1 |
| Valsalva (Before to after Valsalva ratio) | 1.5±0.3 | 1.5±0.2 | 1.8±0.4 | 1.7±0.4 |
| SDNN (ms) | 22 [16 to 26] | 23 [20 to 28] | 39 [29 to 53] | 41 [32 to 54] |
| RMSSD (ms) | 14 [8 to 20] | 14 [10 to 19] | 25 [20 to 39] | 29 [24 to 44] |
| LF (ms^2^) | 34 [16 to 63] | 46 [24 to 67] | 197 [78 to 309] | 207 [123 to 331] |
| HF (ms^2^) | 21 [11 to 44] | 22 [13 to 41] | 71 [46 to 131] | 93 [62 to 180] |
| Total (ms^2^) | 150 [75 to 236] | 179 [126 to 238] | 606 [254 to 1106] | 729 [301 to 1165] |
| LF/HF ratio | 1.8 [0.9 to 3.4] | 1.4 [0.7 to 3.7] | 2.1 [1.4 to 4.2] | 1.9 [1.2 to 4.2] |

Data are n (%), mean±SD, or median [Q1 to Q3]. Albuminuria was defined as a urine albumin excretion rate > 30 mg hours^-1^. 30:15; E:I and Valsalva are heart rate variability response to cardiovascular reflex tests; 30:15 = lying to standing; E:I = expiration to inspiration; Valsalva = The Valsalva maneuver; SDNN = standard deviation of the normal-normal interval; RMSSD = root mean square of the successive differences; LF = low frequency power; HF = high frequency power; Total = total power. *Presence of CAN was defined as two or three pathological cardiovascular autonomic reflex tests.
